# A Virus-Encoded Cell–Cell Fusion Machine Dependent on Surrogate Adhesins

**DOI:** 10.1371/journal.ppat.1000016

**Published:** 2008-03-07

**Authors:** Jayme Salsman, Deniz Top, Christopher Barry, Roy Duncan

**Affiliations:** Department of Microbiology and Immunology, Dalhousie University, Halifax, Nova Scotia, Canada; Mount Sinai School of Medicine, United States of America

## Abstract

The reovirus fusion-associated small transmembrane (FAST) proteins function as virus-encoded cellular fusogens, mediating efficient cell–cell rather than virus–cell membrane fusion. With ectodomains of only ∼20–40 residues, it is unclear how such diminutive viral fusion proteins mediate the initial stages (i.e. membrane contact and close membrane apposition) of the fusion reaction that precede actual membrane merger. We now show that the FAST proteins lack specific receptor-binding activity, and in their natural biological context of promoting cell–cell fusion, rely on cadherins to promote close membrane apposition. The FAST proteins, however, are not specifically reliant on cadherin engagement to mediate membrane apposition as indicated by their ability to efficiently utilize other adhesins in the fusion reaction. Results further indicate that surrogate adhesion proteins that bridge membranes as close as 13 nm apart enhance FAST protein-induced cell–cell fusion, but active actin remodelling is required for maximal fusion activity. The FAST proteins are the first example of membrane fusion proteins that have specifically evolved to function as opportunistic fusogens, designed to exploit and convert naturally occurring adhesion sites into fusion sites. The capacity of surrogate, non-cognate adhesins and active actin remodelling to enhance the cell–cell fusion activity of the FAST proteins are features perfectly suited to the structural and functional evolution of these fusogens as the minimal fusion component of a virus-encoded cellular fusion machine. These results also provide a basis for reconciling the rudimentary structure of the FAST proteins with their capacity to fuse cellular membranes.

## Author Summary

Much of our current understanding of how proteins mediate membrane fusion derives from the study of enveloped virus fusion proteins. These fusion protein complexes function autonomously to co-ordinately regulate virus–cell attachment and subsequent membrane merger. In contrast, the reovirus Fusion-Associated Small Transmembrane (FAST) proteins are the only example of virus-encoded cellular fusogens, specifically designed to mediate cell–cell rather than virus–cell membrane fusion. In view of their small size, it was unclear if, or how, the FAST proteins are responsible for promoting the membrane attachment and close apposition stages of the fusion reaction. We now show that the FAST proteins have specifically evolved to function as the fusion component in a biphasic cell–cell fusion reaction, where the membrane attachment and membrane merger stages represent two distinct, uncoupled phases. Exploiting cadherins as surrogate adhesins, the FAST proteins have retained within their rudimentary structures the minimal determinants required to convert pre-existing adherens junctions into sites of cell–cell membrane fusion. These results raise the interesting possibility that other, yet to be identified cellular fusion proteins may resemble the FAST proteins, using separate adhesins and less complex fusion proteins in a similar biphasic membrane fusion reaction.

## Introduction

By nature of their route of entry into cells, enveloped viruses possess proteins dedicated to the regulation and execution of membrane fusion between the viral envelope and target cell membrane. A multi-step process defines what may be a universal pathway to membrane fusion, involving membrane contact (attachment and enforced close apposition), lipid mixing (hemifusion), and content mixing (pore formation and stabilization) [1]–[3]. Extensive analyses suggest the structural transition of enveloped virus fusion protein complexes from a metastable pre-fusion conformation to a lower energy post-fusion structure provides the energy to drive the multi-step fusion process [4],[5]. Although details of the protein structural rearrangements that accompany membrane fusion have emerged, the precise relationships between structural interactions within components of the fusion machinery and the different steps in the fusion reaction remain unclear.

In spite of considerable diversity in the architecture of the enveloped virus fusion protein complexes, recent studies reveal a remarkable conservation in the relationships between structural remodelling of these protein complexes and the process of membrane merger [6]–[9]. The emerging paradigm predicts that triggered rearrangements in the fusion protein complex result in exposure and membrane insertion of a fusion peptide, followed by folding back of the extended structure and hairpin formation that presumably drives membrane apposition and merger [10],[11]. A second unifying principle is that the enveloped viruses use protein complexes of varying complexity that function autonomously to co-ordinately regulate progression through all stages of the multi-step fusion process. In the simplest situation, the flaviviruses and rhabdoviruses use multiple copies of a single trimeric glycoprotein for the entire fusion reaction [12],[13]. In all other viruses, the activities responsible for membrane attachment and membrane fusion are segregated into different polypeptides or separate multimeric proteins that nonetheless function together as cognate components of an autonomous membrane fusion machine. For example, the ortho-, retro-, filo-, and coronaviruses assign the initial membrane contact and latter membrane merger stages of the process to separate polypeptide subunits within a homotrimeric protein complex [14]–[17]. A slightly different situation occurs in the alphaviruses, where the receptor binding E2 glycoprotein initially forms a heterodimer with the E1 membrane fusion polypeptide; a low pH trigger and insertion of the fusion peptide into target membranes converts E1 to a functional, homotrimeric fusion protein [18]. Even the herpesviruses and the majority of the paramyxoviruses, that utilize separate multimeric proteins for the membrane contact and membrane merger steps of fusion reaction, couple membrane binding to membrane fusion via transient lateral interactions that are believed to be involved in triggering the structural transition of the fusion protein [8],[19]. Coordinating the membrane attachment and membrane fusion stages of the fusion reaction using an autonomous fusion machine reflects the need for enveloped viruses to spatially and temporally regulate fusion of the virus envelope with a suitable target cell membrane.

The concept that all viral fusion proteins function as components of autonomous, metastable fusion machines utilizing extensive structural rearrangements to drive membrane fusion is challenged by the reovirus *f*usion-*a*ssociated *s*mall *t*ransmembrane (FAST) proteins. The FAST proteins are an unusual family of membrane fusion proteins encoded by the fusogenic orthoreoviruses, a diverse group of nonenveloped viruses [20]. At only 95–140 residues in size, the FAST proteins are the smallest known proteins capable of inducing biological membrane fusion. Unlike enveloped virus fusion proteins, the FAST proteins are nonstructural viral proteins and therefore not involved in virus entry into cells [21]. Following their expression inside virus-infected or transfected cells, the FAST proteins traffic to the plasma membrane where their sole defined function is to disseminate the virus infection by mediating membrane fusion with adjacent uninfected cells [22].

The unusual biological role of the FAST proteins is reflected in their unique structural features. There are currently three members of the FAST protein family named according to their approximate molecular masses (p10, p14 and p15), all of which are single-pass transmembrane proteins that assume an N_exoplasmic_/C_cytoplasmic_ membrane topology [20]. Unlike all other fusion machines whose topology orients the majority of the protein on the proximal (i.e. contacting) side of the membrane, as much or more of the mass of the FAST proteins is localized on the distal side of the membrane resulting in small N-terminal ectodomains of only ∼20–40 residues [21],[23],[24]. The FAST proteins also exhibit considerable diversity in their repertoires and arrangement of structural motifs. For example, the p14 and p15 ectodomains contain an essential N-terminal myristate moiety that is lacking in p10, while the p10 and p14 ectodomains have hydrophobic patches that share some similarity to the fusion peptide loops found in class II enveloped virus fusion proteins [25]–[27]; p15 lacks this motif in its ectodomain but has a similar motif in its endodomain [24].

The surprising structural features and diversity of the FAST proteins have not been reconciled with existing models of protein-mediated membrane fusion. The purified p14 FAST protein, when reconstituted into liposome membranes, mediates liposome-cell fusion and liposome-liposome lipid-mixing [28], suggesting the FAST proteins, like the enveloped virus fusion protein complexes, can function as autonomous membrane fusion machines. The FAST protein ectodomains, however, lack the structural complexity typical of most viral fusion proteins and appear to be incapable of using hairpin formation to drive membrane apposition and fusion. If, and how, the FAST proteins mediate the earliest stages of the fusion reaction (i.e. membrane attachment and close apposition) is therefore not apparent.

We now show that although the FAST proteins have retained within their rudimentary structures the activities required to mediate the actual merger of closely apposed membranes, they rely on non-cognate, surrogate receptor-binding proteins to mediate membrane attachment for enhanced cell–cell fusion activity. Furthermore, maximal cell–cell fusion activity requires active actin remodelling. The use of surrogate, non-viral adhesion factors is perfectly suited to the evolution of the FAST proteins as virus-encoded cellular fusogens, and has important implications on mechanistic models of FAST protein-mediated membrane fusion reaction.

## Results

### The p14 FAST protein lacks specific receptor-binding activity

**Figure 1:**
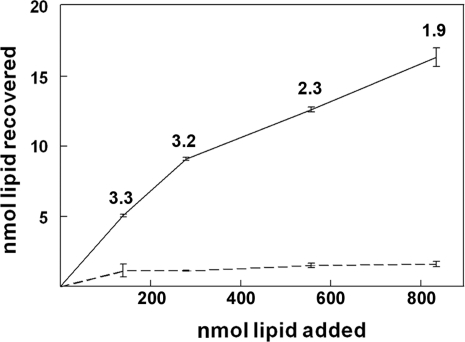
The p14 FAST protein lacks specific receptor-binding activity. Fluorescent liposomes were prepared with (solid line) or without (dashed line) p14, and increasing doses of the liposomes (nmol of lipid) were added to monolayers of QM5 fibroblasts at 4°C. The total nmol of lipid bound were determined by fluorimetry in reference to a standard curve of phospholipid concentrations. Data is presented as the mean±S.D. of a single experiment conducted in triplicate. Numbers within the graph indicate the percent binding efficiency at each input dose of liposomes.

Based on the capacity of the purified p14 FAST protein to induce liposome-cell and liposome-liposome fusion [28], we speculated the FAST proteins are responsible for all stages of the membrane fusion reaction, including the earliest stage of membrane attachment. To directly test this hypothesis, quantitative liposome-cell binding assays were performed. Surprisingly, titration analysis of p14-liposome binding to target cells indicated low-level, non-saturable adherence of p14-liposomes at lipid concentrations exceeding 1.6 mM (Fig. 1). As previously reported [28], p14-liposomes adhered better to target cells than liposomes lacking the p14 FAST protein. However, the percent binding efficiency of p14-liposomes ranged from only 1.9–3.3% of the input liposomes. This low-affinity adherence did not reflect a preponderance of liposomes lacking p14, since flow cytometry revealed the majority of liposomes contained p14, with an average protein density of 6–7×10^3^ p14 molecules per 400 nm liposome [28]. Furthermore, there was no evidence of receptor saturation contributing to the low binding efficiency, as indicated by the progressive increase in bound liposomes with increasing doses of input liposomes (Fig. 1). We conclude that p14 lacks specific high-affinity receptor-binding activity. Therefore, while a low level of non-specific adherence is sufficient to facilitate at least some FAST protein-mediated liposome-cell fusion, these results raised the question of whether surrogate adhesion factors might be essential for, or could enhance, the normal biological functioning of the FAST proteins as cell–cell fusogens.

### Calcium depletion specifically inhibits FAST protein-mediated syncytiogenesis

Since the FAST proteins function as virus-encoded cellular fusogens, we reasoned they could have evolved to specifically rely on cell adhesion proteins to mediate the earliest steps in the fusion reaction. In view of the implications of a multi-component fusion complex on mechanistic models of FAST protein function, we therefore sought to define the relationship, if any, between the FAST proteins and cellular adhesion factors. Cadherins presented as a likely candidate to provide an adhesive activity that could influence FAST protein-mediated cell–cell fusion, since this ubiquitous family of membrane glycoproteins is involved in the formation of virtually all types of homotypic cell–cell adhesion [29]. The broad distribution of cadherin junctions is compatible with the promiscuous cell–cell fusion activity of the FAST proteins.

**Figure 2:**
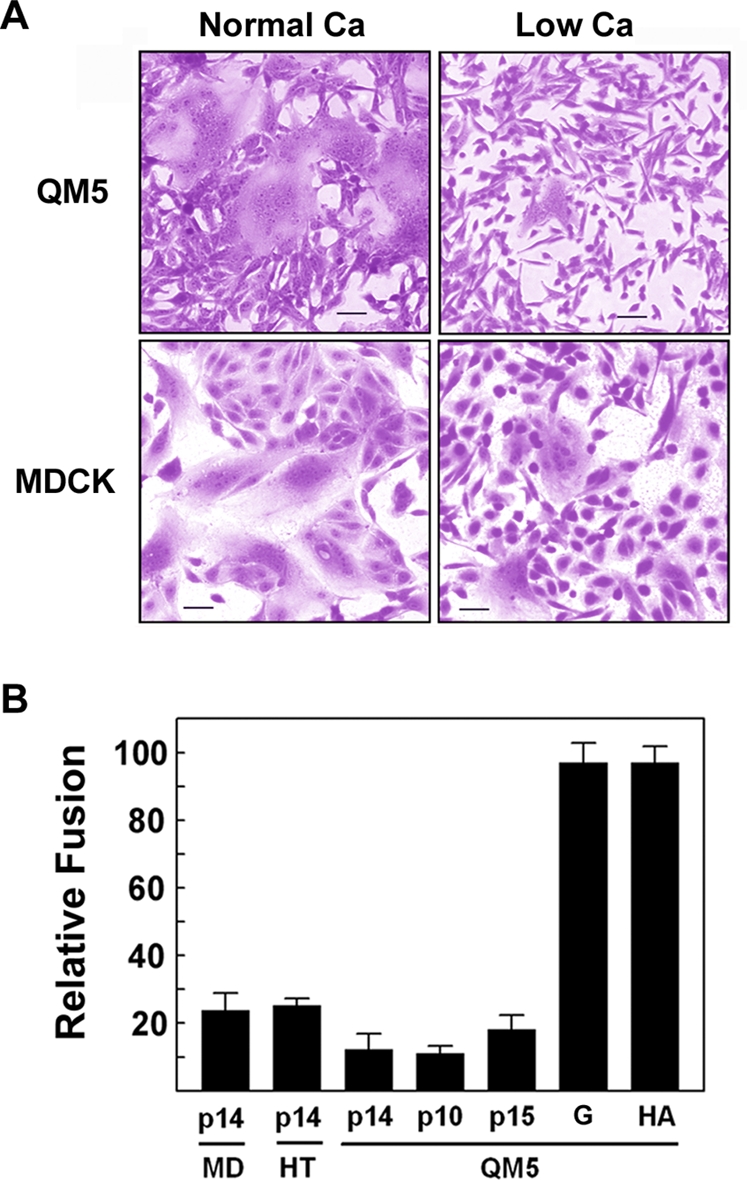
Calcium depletion inhibits FAST protein-mediated syncytiogenesis. (A) QM5 and MDCK cells were transfected with p14, cultured under normal or low calcium conditions, then fixed and Giemsa stained at 8 (QM5) or 20 (MDCK) h post-transfection to detect multinucleated syncytia. Scale bar = 50 µm. (B) Influenza HA- (HA), VSV G- (G) and FAST protein (p10, p14, p15) -transfected QM5 fibroblasts, and p14-transfected MDCK (MD) or HT1080 (HT) epithelial cells were cultured under normal or low calcium conditions. Cells were fixed at various times post-transfection when syncytia were countable, and the average number of syncytial nuclei per field was determined from Giemsa-stained monolayers. Data is presented as the extent of cell fusion under low calcium conditions relative to the same transfected cells cultured for the same length of time under normal calcium conditions, set at 100%. Values represent the mean±S.E. (n = 3). The following numbers are the average syncytial nuclei per field for the different fusogens in the different cell lines under normal calcium levels (i.e. the 100% level): p14 in MDCK cells, 20 h post-transfection - 81.2; p14 in HT1080 cells, 9 h post-transfection - 44.9; p14 in QM5 cells, 7 h post-transfection - 200.4; p10 in QM5 cells, 9 h post-transfection - 83.1; p15 in QM5 cells, 13 h post-transfection - 101.6; VSV-G protein in QM5 cells, 24 h post-transfection, no low pH treatment - 60.9; influenza HA in QM5 cells, low pH treatment at 24 h post-transfection - 293.11.

To initially explore whether cadherins might be involved in the FAST protein-mediated cell–cell fusion reaction, p14-transfected cells were briefly cultured under low calcium conditions prior to, and during, syncytiogenesis. Calcium depletion disrupts homotypic cadherin interactions, resulting in rapid dissociation of cadherin-dependent adhesions without affecting integrin-mediated cell attachment to the substratum [30]. As qualitatively observed by light microscopy of Giemsa-stained monolayers (Fig. 2A), and as quantified using a standard syncytial assay [23] (Fig. 2B), low calcium conditions inhibited p14-induced syncytium formation by 75∼90% in both fibroblast (QM5 and HT-1080) and epithelial (MDCK) cells. The ability of two other members of the FAST protein family, p10 and p15, to fuse QM5 fibroblasts was similarly inhibited by ∼80–90% under low calcium conditions (Fig. 2B). Most importantly, the fusion activity of the vesicular stomatitis virus (VSV) G and influenza HA viral fusion proteins was unaffected by similar low calcium conditions (Fig. 2B); since these enveloped virus fusion proteins have their own receptor-binding activity, their cell–cell fusion activity should be independent of calcium-mediated cadherin contacts. The inhibition of FAST protein-mediated syncytiogenesis under low calcium conditions was therefore unlikely to be due to a generalized inhibitory effect of calcium depletion on cell–cell fusion.

### p14-induced cell–cell membrane fusion is calcium-dependent

**Figure 3:**
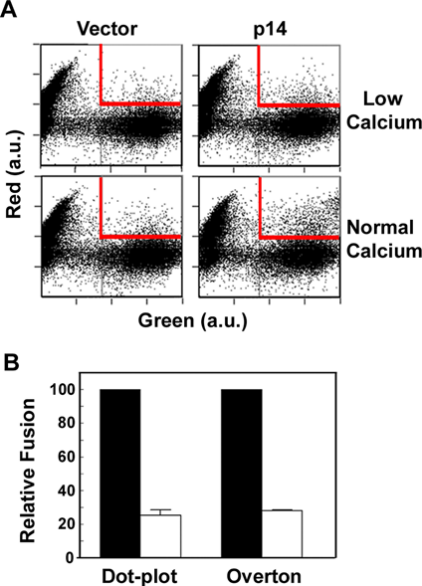
p14-induced cell–cell membrane fusion is calcium-dependent. (A) Two populations of QM5 cells, one labelled with calcein red orange AM (Red) and the other expressing EGFP (Green), were co-cultured, transfected with p14 or empty vector, then cultured under normal or low calcium conditions for 3 h to allow fusion to proceed. Trypsinized, single-cell suspensions were analyzed by flow cytometry, using the increase in cells positive for both green and red fluorophores (gated area shown in red) as an indicator of content mixing. Axes are in arbitrary units (a.u.) of fluorescence intensity. Data is representative of four experiments conducted in duplicate. (B) The gated populations of co-fluorescent p14-transfected cells shown in the dot plots in panel A were quantified, and results are presented as the relative fusion under low (white bars) versus normal (black bars) calcium conditions. Similar analysis was performed using Overton subtractions to quantify the extent of the increase in green fluorescence of the gated red cells.

To determine whether the actual FAST protein-induced membrane fusion reaction, not just syncytiogenesis, was also calcium-dependent, a quantitative pore formation assay [31] was adapted to assess the p14-induced fusion reaction. Two independent populations of cells labelled with either green fluorescent protein or calcein red-orange were seeded together, transfected with a p14 expression plasmid, co-cultured in the absence or presence of calcium to allow cell–cell fusion to proceed, then trypsin-treated to generate a single cell suspension. The number of co-fluorescent cells was then quantified by flow cytometry. Under normal calcium conditions, p14-induced membrane fusion was easily detectible by the increase in the number of co-fluorescent cells relative to the low background level of co-fluorescent cells observed in vector-transfected cells (Fig. 3A). Under low calcium conditions, the transfer of the soluble fluorescent markers in p14-transfected cell monolayers was reduced to near background levels. Quantifying the numbers of co-fluorescent cells in the dot plots from p14-transfected cells indicated that low calcium conditions inhibited the pore formation/expansion stage of membrane fusion by ∼80% (Fig. 3B). Identical results were obtained by quantifying the extent of the increase in green fluorescence of the gated red cells by Overton subtractions (Fig. 3B). These results were highly reproducible over four independent experiments, confirming that both FAST protein-induced syncytium formation and the actual membrane fusion reaction itself are calcium-dependent.

### Cadherins enhance, but are not required, for p14-mediated membrane fusion

**Figure 4:**
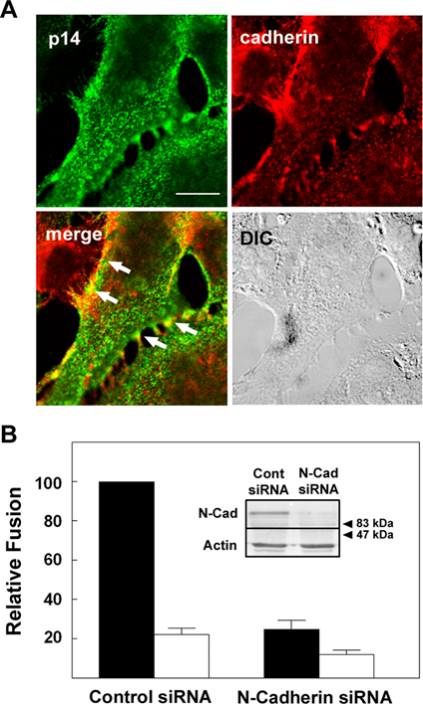
Ablating cadherin expression inhibits p14-mediated syncytiogenesis. (A) Transfected QM5 cells were surface immunostained for p14 (green), then permeabilized and immunostained for N-cadherin (red) at 4 h post-transfection. Arrows in the merged image point to regions of N-cadherin and p14 co-localization at cell–cell contacts as indicated by the yellow pixels. The differential interference microscopy (DIC) image of the same field is shown. Scale bar = 10 µm. (B) HT-1080 cells were transfected with control siRNA or siRNA directed against human N-cadherin, then co-transfected with p14 and cultured under normal (black bars) or low (white bars) calcium conditions. Cells were fixed at 9 h post p14-transfection and syncytiogenesis was quantified by syncytial indexing. Data is presented as the extent of fusion relative to control siRNA-transfected cells cultured under normal calcium conditions. Values represent the mean±S.E. (n = 4). The average number of syncytial nuclei per field under normal calcium levels (i.e. the 100% level) was 53.8. The inset shows Western blot analysis of N-cadherin (N-Cad) and actin expression in the cells transfected with control (Cont) or N-cadherin (N-Cad) siRNA. Numbers indicate the mobilities of M_r_ standards.

If the FAST proteins are relying on cadherins to generate fusion sites, then p14 in the plasma membrane should co-localize at sites of cadherin-mediated adhesion. Immunofluorescence microscopy of transfected QM5 cells revealed the obvious concentration of N-cadherin at sites of cell–cell contact (Fig. 4A). In contrast, p14 was broadly distributed in transfected cells and on the cell surface, with extensive regions of p14 staining that did not overlap with cadherins, suggesting p14 does not specifically localize with cadherins. This conclusion was supported by previous radioimmunoprecipitation studies that did not reveal stable p14-cadherin interactions [23],[25],[32]. There was, however, clear overlap of a percentage of p14 near regions of intense N-cadherin staining (Fig. 4A). Therefore, while p14 does not appear to specifically co-localize with cadherins, the apparently stochastic localization of p14 at sites of cadherin junctions would allow p14 to exploit these junctions for cell–cell fusion.

**Figure 5:**
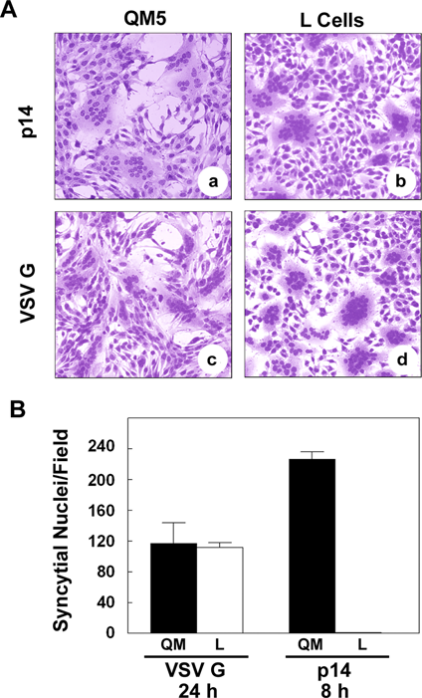
Fusion efficiencies in cadherin-containing and cadherin-deficient cell types. (A) QM5 and L cells were transfected with p14 or VSV G, cultured under normal calcium conditions, then fixed and Giemsa stained at 8 (panel a) or 24 (panels b–d) h post-transfection to detect multinucleated syncytia. Scale bar = 50 µm. (B) QM5 and L cells were transfected with VSV G or p14, cultured under normal calcium conditions, and the average number of syncytial nuclei per field was determined from Giemsa-stained monolayers at the indicated times post-transfection. Values represent the mean±S.D. from a representative of two separate experiments conducted in triplicate.

We also noted a correlation between cadherin status in different cell types and the extent of p14-mediated syncytium formation; this correlation did not apply to the VSV G protein, whose fusion activity is independent of cadherin interactions (Fig. 2B). The VSV G protein is a low pH-activated viral fusion protein that can gradually induce cell–cell fusion when transiently over-expressed in transfected cells in the absence of a triggering acid treatment, due presumably to either gradual acidification of the medium or pH activation of the G protein in the exocytic pathway [33]. Under these conditions, which were chosen since they closely mirror the progressive, untriggered cell–cell fusion mediated by the FAST proteins, VSV G induced equivalent levels of cell–cell fusion by 24 h post-transfection in both cadherin-containing QM5 cells and in cadherin-deficient L cells, as shown qualitatively in Giemsa-stained monolayers (Fig. 5A, panels c and d) and quantitatively by counting syncytial nuclei per field (Fig. 5B). In contrast, p14-induced syncytiogenesis was dramatically different in these two cell types. At 8 h post-transfection, p14 induced extensive syncytium in QM5 cells, as shown qualitatively (Fig. 5A, panel a) and quantitatively (Fig. 5B). A similar situation applied to the cadherin-containing HT1080 cells, which induced extensive syncytium formation by 9 h post-transfection (Fig. 4B). There was no evidence of cell–cell fusion in the p14-transfected L cells at this early timepoint (Fig. 5B), but cell–cell fusion did eventually occur in the L cells, becoming detectible by 17–20 h post-transfection. Even by 24 h post-transfection, however, p14-induced syncytiogenesis in the L cells (Fig. 5A, panel b) was still only ∼20% of that obtained in QM5 cells at 8 h post-transfection. Therefore, although the cadherin-deficient L cells did support p14-induced syncytiogenesis, cell–cell fusion in the L cells was markedly reduced in both the rate and extent of syncytium formation compared to the cadherin-containing QM5 fibroblasts.

**Figure 6:**
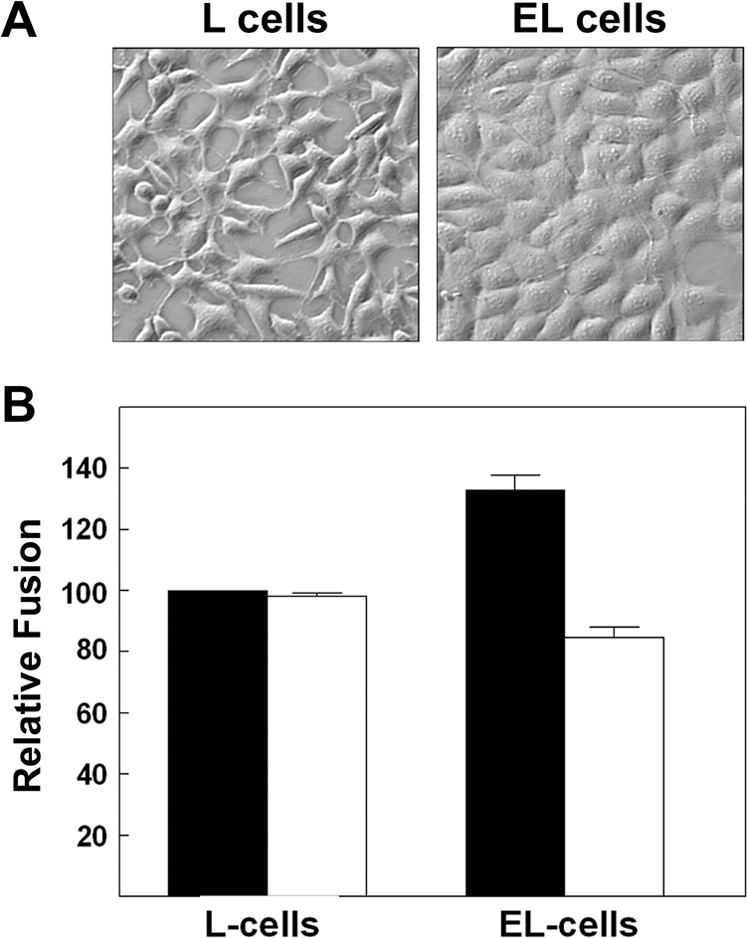
Ectopic cadherin expression enhances p14-mediated fusion. (A) Phase contrast microscopy (400× mag.) of L and EL cells. (B) L and EL cells were transfected with p14, cultured under normal (black bars) or low (white bars) calcium conditions, and the extent of syncytia formation in Giemsa-stained monolayers was quantified at 26 h post-transfection by syncytial indexing. Data is presented as the extent of fusion relative to L cells under normal calcium conditions. Values represent the mean±S.E. (n = 3). The average number of syncytial nuclei per field in the L cells under normal calcium levels (i.e. the 100% level) was 40.7.

Since numerous differences aside from cadherin expression could influence p14-induced syncytiogenesis in QM5 and L cells, two complementary approaches were pursued to more directly examine the influence of cadherins on FAST protein-mediated cell–cell fusion. First, siRNAs were used to silence N-cadherin expression in HT-1080 human fibroblast cells. N-cadherin expression was decreased by >70% in cells transfected with siRNAs directed against human N-cadherin relative to cells transfected with control siRNAs (Fig. 4B). Decreased cadherin expression coincided with a ∼75% decrease in p14-mediated syncytiogenesis, effectively reducing the level of cell–cell fusion to that observed when low extracellular calcium levels were used to disrupt cadherin interactions in control siRNA-transfected HT-1080 cells (Fig. 4B). Second, if reducing cadherin interactions inhibits FAST protein-mediated syncytiogenesis, then would increasing cadherin contacts have the opposite effect? To examine this question, p14-induced syncytium formation was examined in the cadherin-deficient L cells and in EL cells, which are L cells stably expressing E-cadherin [34]. Introduction of E-cadherin into L cell fibroblasts resulted in both a noticeable increase in cell–cell contact (Fig. 6A), and a reproducible 30–40% increase in p14-induced syncytium formation (Fig. 6B). Furthermore, this increase in cell–cell fusion was eliminated when EL cells were cultured under low calcium conditions, suggesting it was directly due to *trans*-cadherin interactions.

The results obtained in the L cells and EL cells, in conjunction with the decrease in FAST protein-induced cell–cell fusion following siRNA knockdown of cadherin expression in HT-1080 cells, clearly indicated that in the absence of their own receptor binding capability, the FAST proteins can exploit cadherin junctions to provide the initial membrane attachment stage of the fusion reaction. However, some level of p14-induced cell–cell fusion persisted under low calcium conditions in fibroblast and epithelial cells (Fig. 2B), in cadherin siRNA-depleted HT1080 cells (Fig. 4B), in L-cells devoid of cadherin (Fig. 5A), and in fusion of p14-liposomes to target cells [28], suggesting cadherins enhance, but are not required, for FAST protein-induced syncytiogenesis.

### The FAST proteins function using diverse surrogate adhesion factors

The apparent lack of specific interactions between p14 and cadherins, coupled with the enhancing though non-essential role of cadherins in the cell–cell fusion reaction mediated by the FAST proteins, suggested cadherins do not represent the cognate membrane attachment component of a bipartite FAST protein fusion complex. Rather, we predicted the FAST proteins evolved as independent membrane fusion proteins that seconded the close membrane apposition stage of the fusion reaction to surrogate, non-cognate adhesins. To test this hypothesis, we examined whether adhesion factors other than cadherins could exert a similar stimulatory effect on the cell–cell fusion activity of the FAST proteins.

**Figure 7:**
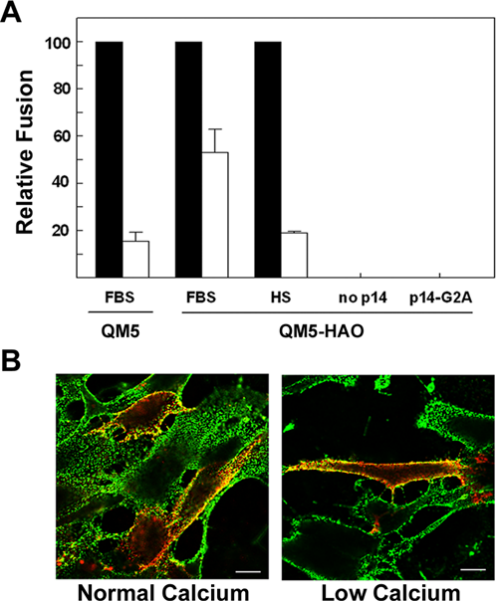
Surrogate receptor binding proteins enhance p14-mediated fusion in the absence of cadherin junctions. (A) QM5 and QM5-HAO cells were transfected with p14 and cell–cell fusion under normal (black bars) or low (white bars) calcium conditions in the presence of FBS was quantified by syncytial indexing at 7 h post-transfection. QM5-HAO cells were also transfected under similar conditions and incubated in the presence of horse serum (HS) to block HAO receptor binding, or were transfected with the non-fusogenic mutant p14-G2A or empty vector (no p14) to assess any contribution of the HAO protein to syncytiogenesis. Data is presented as the extent of fusion under low calcium conditions relative to normal calcium conditions. Values represent the mean±S.E. (n = 3–5). The average numbers of syncytial nuclei per field under normal calcium levels (i.e. the 100% level) were 151.9 (QM5 cells in FBS), 169.4 (QM5-HA0 cells in FBS), and 51.2 (QM5-HA0 cells in HS). (B) Merged surface immunofluorescence microscopy images of epitope-tagged p14 (red) and influenza HA (green) in QM5-HAO cells under normal and low calcium conditions. Yellow indicates regions of p14 and HAO colocalization. Scale bars = 10 µm.

The uncleaved precursor of the influenza virus HA fusion protein, HAO, is fusion-inactive but retains its ability to bind sialic acid. Furthermore, the fusion activity of the cleaved HA protein was unaffected by disrupting cadherin interactions (Fig. 2B). HAO-sialic acid interactions could therefore conceivably substitute for cadherin-mediated cell–cell adhesion under low calcium conditions to enhance FAST protein-induced cell–cell fusion. To explore this possibility, we analyzed p14-induced syncytiogenesis in QM5 cells stably expressing the fusion-inactive influenza HA0 protein. As shown (Fig. 7A), the presence of HAO resulted in a substantial increase in the cell–cell fusion activity of p14 under calcium conditions that disrupt cadherin interactions. The stimulatory effect of HAO on p14-induced cell–cell fusion was ablated using non-immune horse serum (Fig. 7A), which contains α_2_-macroglobulin and other components that inhibit HAO binding to its sialic acid receptor [35], supporting the conclusion that HAO-sialic acid interactions can effectively substitute for cadherin interactions to enhance the p14-induced cell–cell fusion reaction. QM5-HA cells transfected with a non-fusogenic mutant of p14, p14-G2A [23], or with vector alone exhibited no syncytiogenesis (Fig. 7A), confirming that HA0 was not contributing to the fusion reaction beyond providing cell–cell adhesion via its sialic acid binding activity. Furthermore, surface immunofluorescence microscopy revealed that a proportion of p14 co-localized with HAO, including at sites of cell–cell contact, and this colocalization was maintained under low calcium conditions (Fig. 7B). These results supported the hypothesis that the FAST proteins have evolved to retain the minimal activity required to bring about fusion of closely apposed membranes, and rely on non-cognate adhesins to mediate the initial membrane contact phase of the fusion reaction.

### Active adhesion promotes efficient FAST protein-mediated membrane fusion

**Figure 8:**
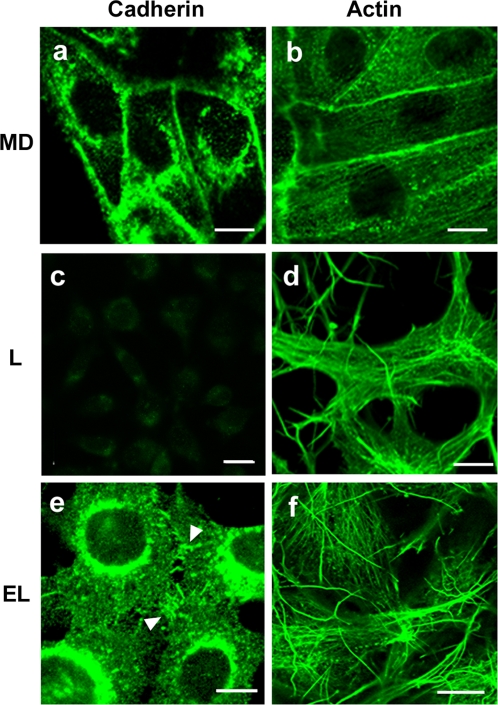
Cadherin and actin distribution in different cell lines. (A) MDCK cells (panels a and b), L cells (panels c and d) and EL cells (panels e and f) were fixed, permeabilized and stained with anti-E-cadherin antibody (left panels) or fluorescently conjugated phalloidin to detect F-actin (right panels). Arrows in panel e point to examples of cadherin junctions at sites of cell–cell contact. Scale bars = 10 µm.

Several features of the surrogate adhesion results suggested that additional factors might be contributing to the syncytiogenic activity of the FAST proteins. First, the enhancing effect of HAO on p14-induced syncytiogenesis did not fully compensate for the loss of cadherin interactions under low calcium conditions (Fig. 7A). Second, although the addition of cadherins increased the susceptibility of L cells to p14-induced fusion, syncytiogenesis in the EL cells was still substantially less than that obtained in QM5 cells. While several explanations could explain these anomalies (e.g. differences in the surface expression of cadherins, FAST proteins, and/or HAO in the different cell lines), one possibility was intracellular events that accompany cadherin interactions. The formation of *trans*-cadherin complexes triggers a cascade of downstream events that convert the weak, focal interactions mediated by individual cadherin pairs to stronger, more extended regions of adhesive contact, a process referred to as “active” adhesion that is intimately dependent on active actin remodelling [36],[37]. During active adhesion, as occurs in MDCK cells (Fig. 8, panels a and b), cadherins and F-actin concentrate at extended regions of close cell–cell contact [38]. In contrast, F-actin was not concentrated at sites of cell–cell contact in cadherin-deficient L cells, forming instead extensive networks of actin fibres (Fig. 8, panels c and d), and these cells did not form extended adhesion contacts, a phenotype we refer to as “no adhesion”. Interestingly, while ectopic expression of E-cadherin in EL cells did result in regions of focal cell–cell contact (Fig. 8, panel e), these focal cadherin junctions did not develop into extended regions of close cell–cell contact. Moreover, the actin cytoskeleton in EL cells retained the architecture observed in L cells, forming an extensive array of stress fibres throughout the cells with little indication of F-actin concentration at sites of cell contact (Fig. 8, panels e and f). This phenotype, where cadherins are engaged but actin is not remodelled to form extended adhesive junctions, has been called “passive adhesion” [37]. There was therefore a correlation between the adhesion properties of the different cell types and their susceptibility to FAST protein-induced cell–cell fusion.

**Figure 9:**
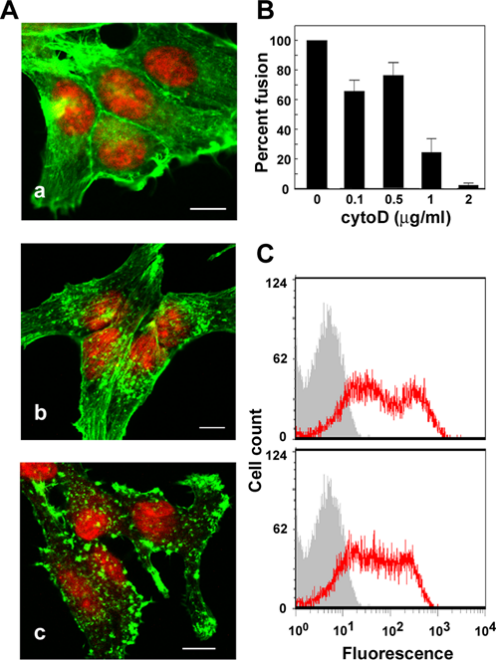
Effects of cytoD on QM5 cell adhesion and p14-induced syncytiogenesis. (A) Fluorescent images of QM5 fibroblasts treated with 0 (a), 0.5 (b) or 1 µg/ml (c) of cytoD for 30 minutes prior to fixation and staining with fluorescently conjugated phalloidin (green) and propidium iodide (red). Scale bars = 10 µm. (B) Cell–cell fusion in QM5 cells treated with the indicated concentrations of cytoD was quantified at 7 h post-transfection by syncytial indexing. Data is presented as the extent of fusion relative to cells untreated with cytoD, set at 100%. Values represent the mean±S.E. (n = 3). (C) QM5 fibroblasts transfected with p14 and left untreated (top panel) or treated with 0.1 µg/ml of cytoD for 1 h (bottom panel) were surface immunostained using an anti-p14 ectodomain-specific antiserum and Alexa Fluor 488-conjugated secondary antibody (red line tracing) and analyzed for cell-surface fluorescence (in arbitrary units) by flow cytometry relative to mock-transfected cells (grey histogram).

To more clearly assess the relationship between active adhesion and syncytium formation induced by the FAST proteins, we sought to generate the three adhesion phenotypes within a single cell type, thereby avoiding potential complications due to possible cadherin-independent differences in different cell lines. Previous studies report that a combination of low calcium conditions and the actin depolymerising agent cytochalasin D (cytoD) can be used to generate the active, passive and no adhesion phenotypes [39]. Following disruption of cadherin contacts by calcium-depletion, reversion to normal calcium conditions in the presence of cytoD allows cadherin engagement but prevents actin polymerization and the formation of the extended regions of cell–cell contact characteristic of active adhesion. A similar procedure was followed to generate these different types of adhesion within the fusion permissive QM5 cells. We first determined the concentration of cytoD that would interfere with actin polymerization in QM5 cells and the formation of new extended regions of cell–cell contact while having minimal inhibitory effects on p14-induced syncytium formation. Low doses of cytoD (0.1–0.5 µg/ml) resulted in the partial redistribution of filamentous actin into cytoplasmic actin aggregates, with minimal effects on stable cadherin junctions (Fig. 9A, panel b) and p14-induced cell–cell fusion (Fig. 9B). The observed 20–30% decrease in syncytium formation in cells treated with low doses of cytoD presumably reflected the previously reported ability of cytoD to disrupt recently formed, unstable cell–cell contacts and prevent the formation of new regions of extended intercellular junctions [38]. The slightly altered distribution in cell surface fluorescence of p14 following cytoD treatment (Fig. 9C) might also have contributed to the modest decline in cell–cell fusion. Increased concentrations of cytoD (above 1 µg/ml) resulted in extensive disruption of the actin cytoskeleton and the formation of cytoplasmic actin aggregates (Fig. 9A, panel c), and inhibited p14-induced syncytium formation by >80% (Fig. 9B). We therefore chose 0.1 µg/ml of cytoD to inhibit the formation of extended cell–cell contacts following calcium depletion and repletion in order to generate the passive adhesion phenotype.

**Figure 10:**
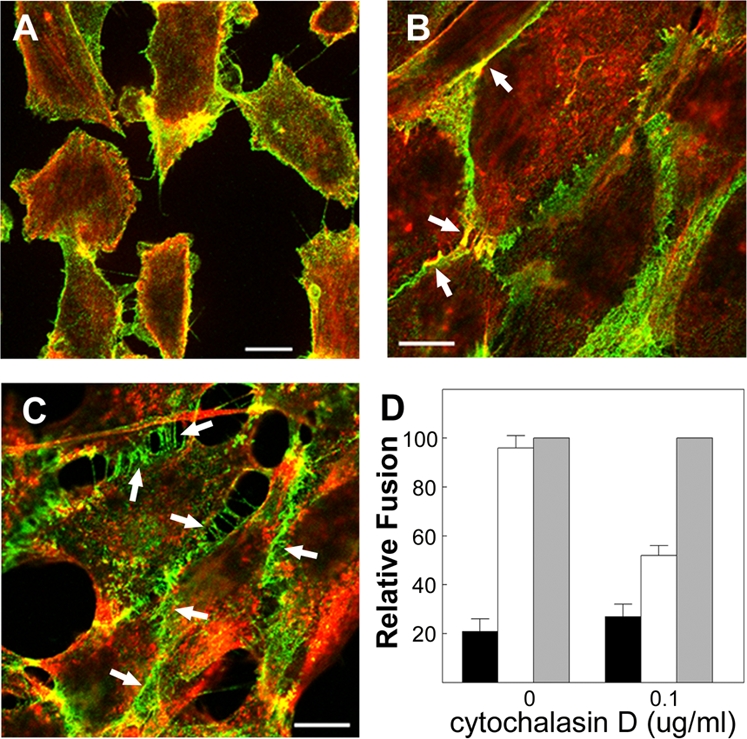
Efficient p14-mediated fusion requires active adhesion. Fluorescent images (panels A–C) of QM5 fibroblasts fixed and stained with anti-N-cadherin antibody (green) or fluorescently conjugated phalloidin (red). Scale bars = 10 µm. (A) Cells cultured under low calcium conditions to disrupt cadherin-mediated cell–cell contacts and generate the no adhesion phenotype. (B) Cells cultured under low calcium conditions and then switched to normal calcium conditions to generate the active adhesion phenotype. (C) Cells cultured under low calcium conditions followed by treatment with cytoD under normal calcium conditions to allow cadherin engagement but not actin remodelling, generating the passive adhesion phenotype. Arrows in (B) indicate examples of F-actin and cadherin colocalization at sites of extended cell–cell contact. Arrows in panel (C) indicate sites of *trans*-cadherin interactions in the absence of extended junction formation. (D) QM5 cells were transfected with p14 and treated to generate the active, passive and no adhesion phenotypes depicted in the previous panels. Just prior to the onset of fusion (4 h post-transfection), cells were briefly subjected to calcium depletion (black and white bars) or maintained in normal calcium levels (grey bars). Immediately after depletion, cells were incubated in normal (white bars) or low calcium (black bars) medium in the presence or absence of cytoD as indicated. The extent of syncytium formation under the different culture conditions was quantified by syncytial indexing 2–4 h after the return to normal calcium containing media. Data is presented as the extent of fusion relative to cells maintained throughout the experiment in normal calcium medium with or without cytoD. Values represent the mean±S.E. (n = 5). The average numbers of syncytial nuclei per field under normal calcium levels (i.e. the 100% level) were 68.9 (no cytochalasin treatment) and 46.9 (with cytochalasin treatment).

Transfected QM5 cells were treated with calcium-free medium for 30 minutes to disrupt cadherin complexes just prior to the onset of p14-induced syncytium formation, and then incubated for a 2–4 h under three different culture conditions to allow cell–cell fusion to progress. First, continued incubation under low calcium conditions to maintain disruption of cadherin-dependent cellular contacts generated the no adhesion phenotype, as evidenced by the loss of extended regions of cadherin-mediated cell–cell contact (Fig. 10A). As previously shown (Fig. 2B), low calcium conditions that generated the no adhesion phenotype inhibited p14-induced cell–cell fusion by ∼80% (Fig. 10D). Second, incubating cells previously cultured in the absence of calcium under normal calcium conditions resulted in the rapid restoration of active adhesion, with cadherin interactions mediating the formation of extended adhesion sites containing both actin and cadherins (Fig. 10B). Syncytiogenesis in these cells was fully restored to the levels observed in cells that were never incubated under low calcium conditions (Fig. 10D). Third, performing the calcium switch in the presence of low concentrations (0.1 µg/ml) of cytoD allowed cadherin contacts to reform but inhibited complete actin remodelling. Under these treatment conditions, cells formed punctate intercellular cadherin contacts (arrows in Fig. 10C), but F-actin was not extensively co-localized in these adhesions and extended cellular junctions did not form, indicative of passive adhesion. This transition of cells from the no-adhesion to passive-adhesion phenotype only partially restored p14-induced syncytium formation to ∼50% of the maximal level (i.e. that observed in cells that were not cultured under low calcium conditions prior to treatment with cytoD). This level of cell–cell fusion closely paralleled the relative fusion efficiency in the QM5-HAO cells under low versus normal calcium conditions (Fig. 7A), conditions that mimic the passive versus active adhesion phenotypes generated by the calcium-switch experiments. Therefore, cells forming active adhesions consistently supported p14-mediated fusion better than cells forming passive adhesions, which in turn fused more efficiently than cells lacking even focal cadherin contacts.

## Discussion

Previous studies revealed that the purified p14 FAST protein is both necessary and sufficient to induce liposome-cell and liposome-liposome fusion [28], suggesting the FAST proteins are autonomous fusion machines responsible for all stages of the membrane fusion reaction. Our present results, however, necessitate a refinement of this general conclusion for the process of FAST protein-mediated cell–cell fusion. We propose that the reovirus FAST proteins contain within their rudimentary structures all of the activities necessary to efficiently mediate the fusion of closely apposed membranes. However, in their natural biological context as cell–cell fusogens, the FAST proteins exploit cellular adhesion factors and active actin remodelling for maximal membrane fusion activity. This is the first such example of a membrane fusion machine comprised of a viral fusion protein that has specifically evolved to utilize surrogate, non-cognate adhesion factors. Two significant implications emerge from this bipartite model. First, the model provides new insights into the form-fits-function evolution of the FAST proteins as virus-encoded cellular fusogens. Second, the use of surrogate, non-cognate adhesins and active actin remodelling provides a means to rationalize the simple structure of the FAST proteins with their role as cellular fusogens.

A prerequisite for all membrane fusion events is the initial tethering of the two membranes to be fused. Enveloped viruses have evolved to utilize a viral adhesin, a component of the viral fusion complex, to mediate cell attachment. Under certain situations, where the viral adhesin is insufficient to provide membrane attachment, surrogate adhesins can provide this activity. For example, the E2 adhesin of the Sindbis virus E1/E2 fusion complex lacks sialic acid binding activity and does not promote virion attachment to red blood cell target membranes. However, co-expression of the influenza virus HAO protein mediates red blood cell attachment to cells expressing E1/E2 and results in efficient cell–cell fusion [3]. There are also instances where, in the absence of suitable receptors on target membranes, the fusion component of the enveloped virus fusion complex can function to promote membrane attachment. This is best exemplified by certain paramyxoviruses whose F protein can function in the absence of the HN attachment component [40], and by fusion of some enveloped viruses to protein-free target liposomes, where membrane attachment is presumably mediated by low pH-triggered exposure of the fusion peptide and insertion into the target membrane [41]–[43]. The above examples underscore the importance of membrane attachment as a prelude to subsequent membrane fusion, and it was therefore not unanticipated that cell–cell fusion mediated by the FAST proteins would also be reliant on membrane attachment. The surprising observation was the discovery that the FAST proteins lack their own adhesion capacity (Fig. 1) and in their natural biological context as cell–cell fusogens, have specifically evolved to use surrogate adhesion factors. Results obtained by calcium depletion (Fig. 2), siRNA knockdown (Fig. 4), and ectopic expression of cadherins in cadherin-deficient cells (Fig. 6) all indicated that cadherins can serve as surrogate adhesins to increase the efficiency of FAST protein-mediated cell–cell fusion. Furthermore, the role of cadherins in enhancing the function of the FAST proteins likely does not reflect a generalized effect of such cellular junctions on cell–cell fusion, as evident by the lack of any adverse effects of disrupting cadherin interactions on syncytiogenesis mediated by two different classes of enveloped virus fusion proteins (Fig. 2), and by the ability of the VSV G protein to induce syncytium formation equally well in both cadherin-containing and cadherin-deficient cell types (Fig 5).

While cadherins can clearly serve as surrogate adhesins for the FAST proteins, there is no evidence that the FAST proteins specifically interact or co-localize with cadherins, and p14 functions to induce liposome-cell fusion with no requirement for cadherins in the donor membrane [28]. Cadherins are therefore not a cognate component of a supramolecular FAST protein fusion machine. The observation that cells are still susceptible to p14-induced syncytium formation in the absence of cadherin-mediated contacts (e.g. cadherin-deficient L cells or in cells whose cadherin contacts are disrupted by calcium depletion or siRNA knockdown) further suggests that other cellular adhesion factors can substitute for cadherins. Nectins, a group of calcium-independent cell adhesion molecules that act upstream of cadherins [44], exhibit weaker interactions than cadherins but occur over a similar distance (i.e. ∼20–25 nm) [45],[46], suggesting they could provide opportunities for the FAST proteins to initiate fusion, albeit with decreased efficiency. Furthermore, HAO receptor binding effectively substituted for cadherin contacts, preserving p14-induced syncytiogenesis to an even greater extent than cadherin-mediated passive adhesion (compare the percent increase in fusion under low versus normal calcium conditions in Figs. 7 and 10). We therefore conclude that the FAST proteins serve as the fusion component of a functionally bipartite fusion machine that is reliant on surrogate, non-cognate adhesion factors to mediate the earliest stages of the fusion reaction. This conclusion further implies that the FAST proteins are not stabilized in a metastable pre-fusion conformation by interactions with their adhesion factors, nor are such interactions involved in triggering the fusion reaction. This is in contrast to the situation with the enveloped virus fusion machines, where spatial relationships between the binding and fusion components frequently influence the folding, stability or triggering of the pre-fusion complex and/or coordinated progression through the fusion reaction [7],[8],[10]. In this biphasic model of FAST protein function, the membrane attachment and membrane merger stages represent two distinct, uncoupled phases. The first phase is mediated by cellular adhesins that do not directly interact with the FAST protein fusogens, which have evolved to function as opportunistic fusogens, retaining within their rudimentary structures all that is needed to complete the second phase by converting naturally occurring adhesion sites into fusion sites.

In addition to the benefits conferred by surrogate adhesion proteins, active adhesion was required for maximal levels of FAST protein-induced cell–cell fusion. Support for this conclusion derives from the reduced cell–cell fusion observed under three different conditions that generated the passive adhesion phenotype; cadherin engagement in the presence of low concentrations of cytoD to partially inhibit actin remodelling (Fig. 10), ectopic expression of cadherins in cadherin-deficient L cells, which did not result in cytoskeletal remodelling (Fig. 8), and the use of HAO as the surrogate adhesin, which does not trigger actin rearrangements (Fig. 7). In contrast, cadherin-mediated actin remodelling did not contribute to the efficacy of cell–cell fusion mediated by either HA or VSV G; these enveloped virus fusion proteins have evolved to function as autonomous fusion machines and both were unaffected by calcium conditions that disrupt cadherin interactions (Fig. 2). The VSV G protein was also equally effective at inducing cell–cell fusion in cadherin-containing QM5 cells and cadherin-deficient L cells (Fig. 5). Although cadherin-mediated actin remodelling was not involved in cell–cell fusion mediated by these enveloped virus fusion proteins, the actin cytoskeleton can affect virus-cell and/or cell–cell fusion when actin dynamics are altered by manipulating the activity of the Rho family GTPases that regulate cytoskeletal structure [47]–[49]. Cytoskeletal remodelling also contributes to extended alignment of the apposing membranes and trafficking of pre-fusion exocytic vesicles to the site of fusion during *Drosophila* myoblast fusion [50]–[52]. As discussed below, numerous changes in the environment of the two contacting membranes that accompany the transition from passive to active adhesion could exert an influence on the FAST protein fusion reaction.

**Figure 11:**
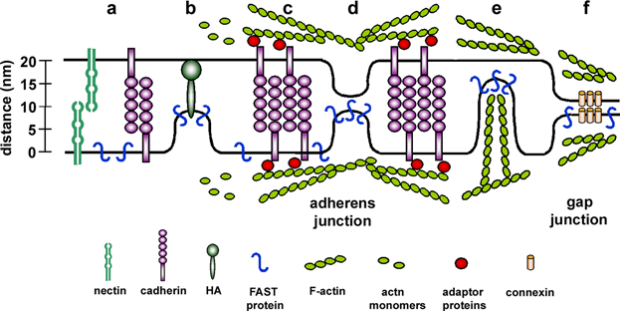
FAST protein-induced cell–cell fusion and surrogate adhesion factors. A model of FAST protein-mediated cell–cell fusion that reflects the small size of the FAST proteins and the possible roles of surrogate receptor binding proteins, actin remodelling, and interbilayer distances in the fusion process. See text for details on steps a–f.

We suggest a model of FAST protein-mediated cell fusion that integrates the unusual structural and functional properties of these fusogens with the enhancing, though non-essential, role of surrogate adhesion factors (Fig. 11). Initial tethering of the two membranes is mediated by either non-specific adhesion of liposomes to target cells, or in the case of cell–cell fusion by weak nectin interactions, passive cadherin engagement or other surrogate adhesins (e.g. HAO). This membrane attachment stage would provide contact sites with interbilayer distances of ∼13–25 nm (Fig. 11, a and b) [29],[45]. These distances are considerably larger than the ∼1.5 nm distance that p14 projects from the membrane in which it resides, as estimated by atomic force microscopy measurements under aqueous conditions and the NMR structure of the p14 ectodomain [25],[32]. In the case of the enveloped viruses, the fusion protein itself is believed to be responsible for breaching this intermembrane distance. A proposed unifying principle for viral protein-mediated membrane fusion involves refolding of the fusion protein from its metastable pre-fusion conformation to its hairpin-like, post-fusion minimal energy state, with mechanical energy serving to pull the membranes into close proximity [11],[53]. Considering the structural limitations of the FAST protein ectodomains, we previously suggested that the FAST proteins are unlikely to adhere to this unifying principle [24],[25],[28]. The present results now provide some alternative possibilities as to how close membrane apposition might be achieved. In the case of passive adhesion, stochastic out-of-plane fluctuations of the membrane (Fig. 11, d) or actin-driven membrane oscillations (Fig. 11, e) as the two apposed membranes “probe” each other could transiently reduce the interbilayer separation to the critical repulsive range of <2–3 nm [4],[54], allowing the FAST proteins to exert their opportunistic fusogenic activity. Active adhesion and the formation of adherens junctions (Fig. 11, c) would increase the probability that membranes reach this critical distance by strengthening weak *trans*-cadherin interactions via lateral clustering of cadherins, by extending the surface area of close cell–cell contact, or by leading to the formation of gap junctions (Fig. 11, f) that reduce intermembrane distances to 2–4 nm [38],[55],[56]. Together, these effects increase the likelihood that suitably stable adhesion sites would exist in close proximity to regions of the plasma membrane containing adequate quantities of the FAST protein needed for fusion. The actin remodelling that accompanies active adhesion could also disrupt cortical actin and/or promote displacement of cellular membrane proteins from the fusion site, both of which can inhibit membrane fusion [5],[57]. While the FAST proteins are unlikely to use hairpin formation to promote close membrane apposition, we do not exclude the possibility that other dynamic structural changes in the FAST proteins could contribute to this process. For example, reversible solvent exposure of hydrophobic residues in the small ectodomain (e.g. amino acids in the ectodomain hydrophobic patch or the N-terminal myristic acid) could alter the hydration layer between membranes while residues in the larger endodomain might contribute to actin remodelling and reductions in intermembrane distances. Studies are currently underway to explore these possibilities.

This biphasic fusion reaction mediated by a viral fusion protein reliant on surrogate non-viral adhesion factors is perfectly suited to the role of the FAST proteins as viral-encoded cellular fusion proteins. As non-structural viral proteins not involved in virus entry, the FAST proteins are not subject to the same spatial and temporal imperatives that dictate the functioning of enveloped virus fusion proteins. Gradual accumulation of the FAST proteins in the plasma membrane of reovirus infected cells, governed by their protein expression from sub-optimal translation start sites and by protein degradation, is all that is required to coordinate the rate of syncytium formation with the virus replication cycle [26],^58^. By exploiting generic adhesion molecules and naturally occurring adhesion junctions, the FAST proteins have the capacity to fuse a diversity of cell types, providing the fusogenic reoviruses with access to the replication machinery of multiple cell types during a single round of replication, leading to rapid localized dissemination of the infection [22]. Seconding the membrane attachment phase of the fusion reaction to surrogate adhesins would also reduce the genetic commitment on the part of the virus, no doubt contributing to the evolution of this remarkable group of fusogenic nonenveloped viruses within the confines of the limited coding capacity of the reovirus genome.

## Materials and Methods

### Cells, antibodies, reagents and clones

Vero and QM5 cells were maintained as previously described [23]. MDCK and HT-1080 cells were maintained in minimal essential medium (MEM) supplemented with 10% fetal bovine serum (FBS). L cells and EL cells were maintained in MEM supplemented with 5% FBS with EL cells also receiving 500 µg/ml G418 to maintain selective pressure [34]. E- and N-cadherin mAbs were from BD Transduction Labs. Goat anti-rabbit and anti-mouse F(Ab)_2_ H+L chain Alexa Fluor 488- and 555-conjugated secondary antibodies and phalloidin were from Molecular Probes. Influenza HA (H1 [WSN]) in pCAGGS and rabbit anti-HA antiserum were a gift from Dr. Earl Brown (University of Ottawa). The actin-disrupting drug cytoD was from Sigma. The p14, p15, p10, p14-G2A and HA epitope-tagged p14 (p14-2HAN) cDNA clones, and the p14 polyclonal and p14 anti-ectodomain antisera were previously described [21],[25],[27],[28]. VSV G protein (Indiana strain) in a eukaryotic expression vector was a gift from Dr. Patrick Lee.

### Liposome binding assay

Fluorescent liposomes and p14-containing proteoliposomes were prepared exactly as previously described [28]. Liposomes (0.4–1.6 mM) were incubated with QM5 fibroblasts on ice for 1 h, then removed and cells were washed with Hank's balanced salt solution (HBSS). The cells were then resuspended in 10 mM EDTA in phosphate-buffered saline (PBS) and bound liposomes were quantified by fluorimetry as previously described [28]. The quantity of lipid molecules bound was calculated using a standard curve comparing fluorescence intensity to phospholipid concentrations.

### Calcium switch assay

Cadherin-mediated cell–cell contacts were disrupted by washing cells with PBS followed by a 1 min incubation with PBS +0.5 mM EDTA, just prior to the onset of syncytiogenesis in the transfected cells. Cells were then washed with PBS and incubated for the duration of the experiment with either MEM or S-MEM (calcium free MEM, Invitrogen) supplemented with 10% dialysed FBS.

### Syncytial indexing

Transfected cells in 12-well cluster plates were fixed with methanol at various times post-transfection based on the extent of syncytium formation in cells incubated under control conditions (e.g. in normal calcium media). Cell–cell fusion was quantified by determining the average number of syncytial nuclei present in five random microscopic fields of Giemsa-stained monolayers, as described previously [23]. Generally, cells were fixed when syncytia in the control wells had progressed to ∼50–250 syncytial nuclei per microscopic field (200× magnification). This level of cell–cell fusion was determined to give the most accurate and reproducible results. For samples with considerably less than an average of 50 syncytial nuclei per field, 10–20 random fields were counted to enhance accuracy. Results are reported as the percent fusion relative to the indicated control treatment, set at 100%.

### FACS-based fusion assay

A population of QM5 cells was labelled with 20 µM calcein red-orange AM (Molecular Probes), mixed 1∶1 with a second population of cells stably expressing EGFP (Clontech) and co-cultured overnight, then transfected with either p14 or control empty vector (pcDNA3). Cells were subjected to calcium depletion at 3 h post-transfection, just prior to the onset of syncytiogenesis, and incubated in either MEM or S-MEM for an additional 3 h. Cells were trypsinized, resuspended in PBS, and analyzed by flow cytometry (FACSCalibur (Becton Dickinson)) using appropriate filter sets and Cell Quest software. A minimum of 300,000 events were recorded, and all data were analyzed using FSC Express 2.0 (De Novo Software).

### RNA silencing

HT-1080 cells were transfected with N-cadherin or control siRNA oligonucleotides (Dharmacon Research Inc.) at a final concentration of 10 nM using INTERFERin transfection reagent (Polyplus Transfection). At 24 h post-transfection, cells were trypsinized and reseeded, cultured for 20 h, then transfected with p14 cDNA using Lipofectamine (Invitrogen). At 3–4 h post-p14 transfection, some wells were depleted of extracellular calcium to disrupt cadherin-mediated contacts as described below. Cells were fixed with methanol at 9 h post-p14 transfection and fusion was quantified by syncytial indexing as described above. Cell lysates were prepared from a parallel experiment and used for Western blot analysis using anti-N cadherin and anti-actin antibodies, HRP-conjugated secondary antibody, and ECL (Amersham Biosciences) according to the manufacturer's instructions, as previously described [32]. Images were captured and quantified using a Typhoon imaging system (Amersham) and ImageQuant software (GE Healthcare).

### Microscopy

Cells grown on gelatin-coated glass coverslips were fixed with 3.7% formaldehyde (20 min) and permeabilized with 0.1% Triton X-100 in PBS (20 min). For surface immunofluorescence, cells were stained as below at 4°C in HBSS prior to fixation with 3.7% formaldehyde. Actin was stained (20 min) with Alexa Fluor 488- or 555-conjugated phalloidin. N- and E-cadherin, HA, p14 and p14-2HAN were detected by incubating cells for 1 h with the appropriate primary antibody followed by fluorophore-conjugated secondary antibodies for 45 min. Cells were mounted with fluorescent mounting medium (Dako), images were acquired with LSM imaging software on a Zeiss LSM510 META laser scanning confocal microscope using the 488 nm argon laser for Alexa Fluor 488 or the 548 nm HeNe laser for Alexa Fluor 555. Images were captured with the 63× or 100× Plan APOCHROMAT (1.4 NA) objective lenses and processed in Adobe Photoshop version 6.0 using only linear adjustments.

### Active vs. passive adhesion assay

Three different adhesion phenotypes (active, passive, no adhesion) were generated in p14-transfected QM5 cells using a modified protocol [39]. Active adhesion was obtained by culturing cells in growth medium containing normal calcium levels with or without 0.1 µg/ml cytoD, or by culturing cells for 30 min in medium lacking calcium followed by incubation in growth media with normal calcium. No adhesion was generated by calcium depletion followed by continued incubation in calcium-free media in the presence and absence of 0.1 µg/ml cytoD. Passive adhesion was generated by first subjecting cells to calcium depletion to disrupt cellular junctions, followed by incubation in normal calcium containing medium containing 0.1 µg/ml cytoD. These conditions allowed cadherin-mediated contacts to form, but inhibited actin remodelling and the development of extended junction formation. Cells under all three conditions were fixed 2–4 h after the calcium switch and processed either for fluorescent microscopy or for quantification of fusion as described above.

### HA and VSV G fusion assay

QM5 cells stably expressing influenza HAO (QM5-HAO) were selected using G418 (Gibco), and HAO expression was confirmed by immunostaining. Cleavage of the HA0 precursor to its fusion-active HA form was accomplished by treatment with 10 µg/ml of trypsin for 5 min in HBSS. Cells were then washed and incubated in growth media containing 10% FBS for 10 min to inhibit residual trypsin activity. When appropriate, calcium was depleted using the calcium switch assay described above, followed by incubation for 20 min in MEM or S-MEM+10% dFBS to allow HAO or HA receptor interactions to form in low calcium conditions. Fusion was triggered with MEM or S-MEM at pH 4.8 containing 10 mM citrate buffer for 1 min. Cells were then transferred to MEM or S-MEM with 10% dFBS to allow syncytia to progress (20–40 min), then fixed with methanol, Giemsa-stained and syncytia were quantified as described above. For VSV-G fusion, cells were transiently transfected and cell–cell fusion was allowed to gradually progress without a specific low pH treatment to activate fusion, as previously reported [32]. This situation more closely mirrors the untriggered cell–cell fusion mediated by the FAST proteins.

## References

[1] Kemble GW, Danieli T, White JM (1994). Lipid-anchored influenza hemagglutinin promotes hemifusion, not complete fusion.. Cell.

[2] Jahn R, Lang T, Sudhof TC (2003). Membrane fusion.. Cell.

[3] Zaitseva E, Mittal A, Griffin DE, Chernomordik LV (2005). Class II fusion protein of alphaviruses drives membrane fusion through the same pathway as class I proteins.. J Cell Biol.

[4] Chernomordik LV, Kozlov MM (2005). Membrane hemifusion: crossing a chasm in two leaps.. Cell.

[5] Chernomordik LV, Zimmerberg J, Kozlov MM (2006). Membranes of the world unite!. J Cell Biol.

[6] Lamb RA, Paterson RG, Jardetzky TS (2006). Paramyxovirus membrane fusion: lessons from the F and HN atomic structures.. Virology.

[7] Kielian M, Rey FA (2006). Virus membrane-fusion proteins: more than one way to make a hairpin.. Nat Rev Microbiol.

[8] Rey FA (2006). Molecular gymnastics at the herpesvirus surface.. EMBO Rep.

[9] Stiasny K, Heinz FX (2006). Flavivirus membrane fusion.. J Gen Virol.

[10] Earp LJ, Delos SE, Park HE, White JM (2005). The many mechanisms of viral membrane fusion proteins.. Curr Top Microbiol Immunol.

[11] Harrison SC (2005). Mechanism of membrane fusion by viral envelope proteins.. Adv Virus Res.

[12] Roche S, Rey FA, Gaudin Y, Bressanelli S (2007). Structure of the prefusion form of the vesicular stomatitis virus glycoprotein G.. Science.

[13] Stiasny K, Kossl C, Lepault J, Rey FA, Heinz FX (2007). Characterization of a structural intermediate of flavivirus membrane fusion.. PLoS Pathog.

[14] Wilson IA, Skehel JJ, Wiley DC (1981). Structure of the haemagglutinin membrane glycoprotein of influenza virus at 3 A resolution.. Nature.

[15] Xu Y, Liu Y, Lou Z, Qin L, Li X (2004). Structural basis for coronavirus-mediated membrane fusion. Crystal structure of mouse hepatitis virus spike protein fusion core.. J Biol Chem.

[16] Weissenhorn W, Carfi A, Lee KH, Skehel JJ, Wiley DC (1998). Crystal structure of the Ebola virus membrane fusion subunit, GP2, from the envelope glycoprotein ectodomain.. Mol Cell.

[17] Weissenhorn W, Dessen A, Harrison SC, Skehel JJ, Wiley DC (1997). Atomic structure of the ectodomain from HIV-1 gp41.. Nature.

[18] Gibbons DL, Vaney MC, Roussel A, Vigouroux A, Reilly B (2004). Conformational change and protein-protein interactions of the fusion protein of Semliki Forest virus.. Nature.

[19] Bagai S, Lamb RA (1995). Quantitative measurement of paramyxovirus fusion: differences in requirements of glycoproteins between simian virus 5 and human parainfluenza virus 3 or Newcastle disease virus.. J Virol.

[20] Duncan R, Corcoran J, Shou J, Stoltz D (2004). Reptilian reovirus: a new fusogenic orthoreovirus species.. Virology.

[21] Shmulevitz M, Duncan R (2000). A new class of fusion-associated small transmembrane (FAST) proteins encoded by the non-enveloped fusogenic reoviruses.. Embo J.

[22] Salsman J, Top D, Boutilier J, Duncan R (2005). Extensive syncytium formation mediated by the reovirus FAST proteins triggers apoptosis-induced membrane instability.. J Virol.

[23] Corcoran JA, Duncan R (2004). Reptilian reovirus utilizes a small type III protein with an external myristylated amino terminus to mediate cell-cell fusion.. J Virol.

[24] Dawe S, Corcoran JA, Clancy EK, Salsman J, Duncan R (2005). Unusual topological arrangement of structural motifs in the baboon reovirus fusion-associated small transmembrane protein.. J Virol.

[25] Corcoran JA, Syvitski R, Top D, Epand RM, Epand RF (2004). Myristoylation, a protruding loop, and structural plasticity are essential features of a nonenveloped virus fusion peptide motif.. J Biol Chem.

[26] Shmulevitz M, Epand RF, Epand RM, Duncan R (2004). Structural and functional properties of an unusual internal fusion peptide in a nonenveloped virus membrane fusion protein.. J Virol.

[27] Dawe S, Duncan R (2002). The S4 genome segment of baboon reovirus is bicistronic and encodes a novel fusion-associated small transmembrane protein.. J Virol.

[28] Top D, de Antueno R, Salsman J, Corcoran J, Mader J (2005). Liposome reconstitution of a minimal protein-mediated membrane fusion machine.. Embo J.

[29] Koch AW, Manzur KL, Shan W (2004). Structure-based models of cadherin-mediated cell adhesion: the evolution continues.. Cell Mol Life Sci.

[30] Volberg T, Geiger B, Kartenbeck J, Franke WW (1986). Changes in membrane-microfilament interaction in intercellular adherens junctions upon removal of extracellular Ca2+ ions.. J Cell Biol.

[31] Lin X, Derdeyn CA, Blumenthal R, West J, Hunter E (2003). Progressive truncations C terminal to the membrane-spanning domain of simian immunodeficiency virus Env reduce fusogenicity and increase concentration dependence of Env for fusion.. J Virol.

[32] Corcoran JA, Salsman J, de Antueno R, Touhami A, Jericho MH (2006). The p14 fusion-associated small transmembrane (FAST) protein effects membrane fusion from a subset of membrane microdomains.. J Biol Chem.

[33] Roberts PC, Kipperman T, Compans RW (1999). Vesicular stomatitis virus G protein acquires pH-independent fusion activity during transport in a polarized endometrial cell line.. J Virol.

[34] Nagafuchi A, Shirayoshi Y, Okazaki K, Yasuda K, Takeichi M (1987). Transformation of cell adhesion properties by exogenously introduced E-cadherin cDNA.. Nature.

[35] Ryan-Poirier KA, Kawaoka Y (1991). Distinct glycoprotein inhibitors of influenza A virus in different animal sera.. J Virol.

[36] Perez-Moreno M, Jamora C, Fuchs E (2003). Sticky business: orchestrating cellular signals at adherens junctions.. Cell.

[37] Vasioukhin V, Fuchs E (2001). Actin dynamics and cell-cell adhesion in epithelia.. Curr Opin Cell Biol.

[38] Adams CL, Chen YT, Smith SJ, Nelson WJ (1998). Mechanisms of epithelial cell-cell adhesion and cell compaction revealed by high-resolution tracking of E-cadherin-green fluorescent protein.. J Cell Biol.

[39] Vasioukhin V, Bauer C, Yin M, Fuchs E (2000). Directed actin polymerization is the driving force for epithelial cell-cell adhesion.. Cell.

[40] Paterson RG, Russell CJ, Lamb RA (2000). Fusion protein of the paramyxovirus SV5: destabilizing and stabilizing mutants of fusion activation.. Virology.

[41] Stegmann T, Delfino JM, Richards FM, Helenius A (1991). The HA2 subunit of influenza hemagglutinin inserts into the target membrane prior to fusion.. J Biol Chem.

[42] White J, Helenius A (1980). pH-dependent fusion between the Semliki Forest virus membrane and liposomes.. Proc Natl Acad Sci U S A.

[43] Corver J, Ortiz A, Allison SL, Schalich J, Heinz FX (2000). Membrane fusion activity of tick-borne encephalitis virus and recombinant subviral particles in a liposomal model system.. Virology.

[44] Irie K, Shimizu K, Sakisaka T, Ikeda W, Takai Y (2004). Roles and modes of action of nectins in cell-cell adhesion.. Semin Cell Dev Biol.

[45] Zhu B, Chappuis-Flament S, Wong E, Jensen IE, Gumbiner BM (2003). Functional analysis of the structural basis of homophilic cadherin adhesion.. Biophys J.

[46] Dong X, Xu F, Gong Y, Gao J, Lin P (2006). Crystal structure of the V domain of human Nectin-like molecule-1/Syncam3/Tsll1/Igsf4b, a neural tissue-specific immunoglobulin-like cell-cell adhesion molecule.. J Biol Chem.

[47] Gower TL, Pastey MK, Peeples ME, Collins PL, McCurdy LH (2005). RhoA signaling is required for respiratory syncytial virus-induced syncytium formation and filamentous virion morphology.. J Virol.

[48] Pontow SE, Heyden NV, Wei S, Ratner L (2004). Actin cytoskeletal reorganizations and coreceptor-mediated activation of rac during human immunodeficiency virus-induced cell fusion.. J Virol.

[49] Schowalter RM, Wurth MA, Aguilar HC, Lee B, Moncman CL (2006). Rho GTPase activity modulates paramyxovirus fusion protein-mediated cell-cell fusion.. Virology.

[50] Chen EH, Olson EN (2005). Unveiling the mechanisms of cell-cell fusion.. Science.

[51] Chen EH, Grote E, Mohler W, Vignery A (2007). Cell-cell fusion.. FEBS Lett.

[52] Kim S, Shilagardi K, Zhang S, Hong SN, Sens KL (2007). A critical function for the actin cytoskeleton in targeted exocytosis of prefusion vesicles during myoblast fusion.. Dev Cell.

[53] Park HE, Gruenke JA, White JM (2003). Leash in the groove mechanism of membrane fusion.. Nat Struct Biol.

[54] Cohen FS, Melikyan GB (2004). The energetics of membrane fusion from binding, through hemifusion, pore formation, and pore enlargement.. J Membr Biol.

[55] Chu YS, Thomas WA, Eder O, Pincet F, Perez E (2004). Force measurements in E-cadherin-mediated cell doublets reveal rapid adhesion strengthened by actin cytoskeleton remodeling through Rac and Cdc42.. J Cell Biol.

[56] Sosinsky GE, Nicholson BJ (2005). Structural organization of gap junction channels.. Biochim Biophys Acta.

[57] Eitzen G (2003). Actin remodeling to facilitate membrane fusion.. Biochim Biophys Acta.

[58] Shmulevitz M, Corcoran J, Salsman J, Duncan R (2004). Cell-cell fusion induced by the avian reovirus membrane fusion protein is regulated by protein degradation.. J Virol.

